# Comparative Proteomics Analysis Reveals Differential Immune Responses of *Paralichthys olivaceus* to *Edwardsiella tarda* Infection Under High and Low Temperature

**DOI:** 10.3390/biology14101417

**Published:** 2025-10-15

**Authors:** Xiaojuan Chen, Lejia Luo, Beibei Zhang, Xiaowei Zhou, Kaipeng Zhang, Panpan Zhang, Bin Sun

**Affiliations:** 1 State Key Laboratory of Mariculture Breeding, Key Laboratory of Marine Biotechnology of Fujian Province, College of Marine Sciences, Fujian Agriculture and Forestry University, Fuzhou 350002, China; cxj@fafu.edu.cn (X.C.); luolejia@163.com (L.L.); zxwdyx1206@163.com (X.Z.); 17611544333@163.com (K.Z.); 2 Institute of Biotechnology, Fujian Academy of Agricultural Sciences, Fuzhou 350003, China; 3 College of Animal Science and Technology, Qingdao Agricultural University, Qingdao 266109, China; 4 CAS Key Laboratory of Experimental Marine Biology, Institute of Oceanology, Center for Ocean Mega-Science, Chinese Academy of Sciences, Qingdao 266071, China; 5 State Key Laboratory of Marine Resource Utilization in South China Sea, Hainan University, Haikou 570228, China; 15208989481@163.com

**Keywords:** *Edwardsiella tarda*, temperature changes, *Paralichthys olivaceus*, immune response, proteomics

## Abstract

**Simple Summary:**

The health of farmed marine fish, including the Japanese flounder (*Paralichthys olivaceus*), is severely compromised by temperature fluctuations and infections such as *Edwardsiella tarda*, resulting in significant economic losses. A previous study revealed that elevated temperature enhanced *E. tarda* dissemination in flounder tissues. However, the underlying mechanism has not been fully explained, especially the changes in protein level. This study utilized label-free proteomics to investigate the immune response of flounder to *E. tarda* infection under low (15 °C) and high (23 °C) temperature conditions. Multiple differentially abundant proteins (DAPs) were identified in each group. GO and KEGG analyses highlighted immune-related pathways and proteins, with key DAPs forming extensive interaction networks. Venn analysis revealed distinct responses: high temperature promoted endocytosis and complement activation, while low temperature increased histone levels and impaired RNA transport. These results enhance our understanding of how water temperature changes affect antibacterial immunity in fish.

**Abstract:**

Fluctuating water temperatures and bacterial pathogens such as *Edwardsiella tarda* pose a serious threat to mariculture, resulting in significant economic losses within the flounder industry. A previous study revealed that elevated temperature enhanced *E. tarda* dissemination in flounder tissues. However, the underlying mechanism has not been fully explained, especially the changes in protein level. In this study, label-free proteomics was utilized to investigate the impact of high temperature (23 °C) and low temperature (15 °C) on flounder immune response to *E. tarda* infection. Our results identified 317 differentially abundant proteins (DAPs) in the low-temperature group (LI-LC) and 302 DAPs in the high-temperature group (HI-HC). GO and KEGG analyses of DAPs revealed numerous immune-related proteins and pathways. Twenty-six key DAPs in the LI-LC group and twenty-seven key DAPs in the HI-HC group were further identified and formed extensive interaction networks, respectively. Through the analysis of key immune-related DAPs that were specifically identified in both groups via Venn diagram analysis, we demonstrated that the endocytosis capacity and complement activity were enhanced in the HI-HC group, while histone abundance and RNA transport function were, respectively, increased and severely interfered with in the LI-LC group. These findings highlight a clear divergence in the immune response of flounder to *E. tarda* infection between 15 °C and 23 °C, providing valuable insights into how temperature variation influences antibacterial immunity in fish.

## 1. Introduction

Water temperature is one of the most crucial environmental factors that affect fish growth [1], sex differentiation [2], metabolism [3], and immunity [4]. Previous studies have demonstrated that low temperatures can inhibit host immune responses, and the degree of immunosuppression is influenced by both the extent and duration of low temperature [5,6,7,8]. For example, the complement activity of sockeye salmon (*Oncorhynchus nerka*) reared at 8 °C was comparable to that measured in fish maintained at 12 °C [5]. However, tilapia (*Oreochromis mossabicus*) reared at 19 °C showed a lower complement activity than at 27 °C [6]. In addition, a previous study examined the short-term cold exposure of tilapia (17 °C for 30 min) and found that no difference in complement activity was observed compared to fish maintained at 27 °C [7]. In contrast, rainbow trout maintained at 5 °C for over two months exhibited significantly reduced complement activity compared to fish maintained at temperatures above 10 °C [8]. Therefore, both the extent and duration of low temperature obviously influenced the degree of immunosuppression. Furthermore, the low temperature was also observed to suppress the production of antibodies [9,10]. On the other hand, high temperatures have been shown to exert complex effects on immune function across different fish species. For instance, a study on tilapia revealed that lysozyme activity decreased at high temperature [6]. Comparable results were also observed on Japanese flounder, where plasma lysozyme activity and IgM concentration were significantly inhibited at high temperature [11]. In Nile tilapia, the IgM concentration was observed to decrease at high temperature [12]. While these three mentioned studies reported suppressive effects of high temperature on fish immunity, others have documented limited effects [13,14] or even beneficial outcomes [8,15]. These discrepancies may arise from factors such as the rate of temperature increase (acute/chronic exposure), temperature setting, experiment duration (short/long term), and inherent differences among fish species. Therefore, it remains unclear whether high temperatures exert beneficial or suppressive effects on fish immunity, as current evidence shows considerable variation across studies.

However, during fish disease outbreaks, changes in water temperature and pathogen invasion often occur simultaneously, resulting in more complex immune responses of the host. Previous studies have demonstrated that temperature significantly influences the expression of immune-related genes in fish [16,17]. In rainbow trout infected with *Yersinia ruckeri*, qRT-PCR analysis revealed that the expression of immune-related genes (interleukin-10, interleukin 1β, interferon γ) was more highly up-regulated at 25 °C compared to 5 °C [16]. In Japanese flounder, the researchers used qRT-PCR to evaluate the expression level of immune-related genes under both temperature changes (15 °C and 22 °C) and inactived *Edwardsiella tarda* infection, and the results showed that the genes belonging to the IFNγ signaling pathway were upregulated at 22 °C but not at 15 °C [17]. However, transcriptional changes may not be entirely consistent with changes in protein levels, and the inactive *E. tarda* is different from live *E. tarda*, which has developed immune escape strategies to avoid host killing. Therefore, in this study, we employed label-free proteomics to investigate the effects of high temperature (23 °C) and low temperature (15 °C) on flounder immune response to live *E. tarda* infection.

Japanese flounder is an economically important fish species in China and has been reported to be susceptible to *E. tarda* infection [18,19]. *E. tarda* is a Gram-negative bacterium that can infect both humans and fish species [18]. The pathogenicity of *E. tarda* relies on various virulence factors, including the type III secretion system (T3SS), the type VI secretion system (T6SS), and other virulence-associated factors [19]. Temperature plays a critical role in regulating the virulence of *E. tarda*, and the sensing of environmental temperature fluctuations is mediated by the two-component system PhoP-PhoQ, in which the sensor histidine kinase PhoQ phosphorylates the response regulator PhoP and activates the expression of T3SS, T6SS, and other virulence genes, thereby facilitating the incidence of edwardsiellosis [20]. In Japanese flounder, *E. tarda* infection often results in a systemic disease known as edwardsiellosis, which is characterized by symptoms such as ascites, hernia, and severe lesions of the internal organs (spleen, kidney, liver, etc.), and has caused significant economic losses in the large-scale farming of flounders [18]. The optimal growth temperature range for flounder is 15–25 °C [21,22], and the outbreak of Edwardsiellosis caused by *E. tarda* usually occurs under imbalanced environmental conditions, such as temperature changes [23]. Therefore, in this study, water temperature conditions were set at 15 °C and 23 °C to reveal the differences in the protein abundance of flounders in response to *E. tarda* infection.

Proteomics is regarded as an efficient means for comprehensive analysis of proteins in a biological system with high throughput. Compared to conventional transcriptomics, proteomics provides a more direct demonstration of host responding differences in protein level under various stimulations [24]. In this study, label-free proteomics were applied to investigate the effects of high and low temperatures on the immune response of flounder infected with *E. tarda*. A large number of differentially abundant proteins have been identified, and a Venn diagram was employed to distinguish unique DAPs between the two groups, with particular focus on those associated with immune responses. Our results provide valuable insights for further research on how temperature fluctuations affect antibacterial immunity in fish.

## 2. Materials and Methods

### 2.1. Ethics Statement

The animal study was reviewed and approved by the Ethics Committee of Fujian Agriculture and Forestry University (Approval number: CEREAW20240189. Date: 12 February 2024).

### 2.2. Japanese Flounder

Japanese flounder weighing approximately 250 g were obtained from a fish farm in Qingdao, China. The fish were kept in laboratory aquariums and were fed a daily diet of commercial food as previously described [25]. Prior to the start of experimentation, the flounders were verified to be healthy through plating count analysis as previously reported [26]. Briefly, fish were randomly selected, and samples of blood, liver, kidney, and spleen were collected to screen for common bacterial pathogens such as *E. tarda*, *Vibrio harveyi*, *Vibrio anguillarum*, *Pseudomonas fluorescens*, and others. For bacterial detection, tissues were homogenized in PBS, and both homogenates and blood were plated in triplicate onto LB agar plates. After incubation at 28 °C for 48–72 h, no bacterial growth was observed in the examined fish samples, confirming the fish to be healthy. To collect spleen tissues, the flounders were humanely euthanized with tricaine methane sulfonate (MS-222) (Sigma, St. Louis, MO, USA) to minimize the suffering of the fish. Briefly, the MS-222 solution was prepared at a concentration of 200 mg/L and buffered with 300 mg/L NaHCO_3_. Fish were immersed in the solution for 20 min to ensure irreversible euthanasia. Euthanasia was confirmed by the loss of motor function, complete cessation of opercular movement, and absence of response to strong external stimuli. Immediately after confirmation, the fish were dissected on ice for spleen collection. For the feeding regime for the experimental fish, fish were fed regularly three times daily at 6:00, 11:00, and 16:00. Fish were hand-fed, and the daily ration was set at 2% of the total fish body weight. In addition, fish were kept in a recirculating aquaculture system in the laboratory. The recirculating aquaculture system laboratory utilized temperature monitoring equipment to achieve precise temperature control. The salinity was maintained within the range of 31–32‰, and the dissolved oxygen level in the water remained above 6.0 mg/L throughout the entire experimental period. Water was changed twice a day (morning and evening), and water quality was monitored once daily.

### 2.3. In Vivo Infection Experiment of Flounder and Samples Collection

A total of 36 flounders were randomly divided into four groups (9 fish/group), including the low-temperature control group (LC), the low-temperature infection group (LI), the high-temperature control group (HC), and the high-temperature infection group (HI). The LC and LI groups were maintained continuously at 15 °C, while the water temperature in the HC and HI groups gradually increased at a rate of 1 °C per day until it reached 23 °C. Afterwards, flounders adapted to the temperature for one week. The bacterial strain used in this study was *Edwardsiella tarda* TX1, which was originally isolated from naturally diseased flounder as previously reported [27] and is not a standardized reference strain. *E. tarda* was cultured in LB medium at 28 °C to an OD_600_ of 0.7. The bacteria were then collected by centrifugation at 8000× *g* for 8 min. Before the bacterial stimulation, the LD50 of *E. tarda* was assessed and found to be 1 × 10^7^ colony-forming units (CFU) in flounder. For the actual experiment, each flounder in the experimental group was injected with 1 × 10^8^ CFU *E. tarda* in a 100 μL volume, and thus the challenging concentration of *E. tarda* was 1 × 10^8^ CFU/mL. Subsequently, bacterial pellets were washed and suspended with 1 × PBS to a final concentration of 1 × 10^8^ CFU/mL. The experimental groups (LI, HI) were intramuscularly injected with 100 μL bacterial suspension per fish, while the control group (LC, HC) was injected with the same volume of 1 × PBS. At 24 h post-infection, the spleen tissues from each group were aseptically obtained. For proteomics analysis, spleen tissues from three fish in each group were pooled to form a single biological replicate. Each group included three such independently pooled replicates (N = 3), resulting in a total of twelve pooled samples across the four groups (LC, HC, LI, and HI). This pooling strategy was adopted to obtain a representative average response at the group level, reduce inter-individual biological variability, and fulfill the minimum tissue quantity required for subsequent sequencing and functional assays.

### 2.4. Sample Preparation for Proteomics and Data Analysis

For sample preparation and protein digestion, a total of twelve spleen samples were collected from the low-temperature group and the high-temperature group. Before the protein digestion, each sample was first cracked with SDT buffer (4% SDS, 100 mM Tris-HCl, 1 mM DTT, pH7.6). The protein concentration was then quantified by the BCA Protein Assay Kit (Bio-Rad, Hercules, CA, USA). Protein digestion was performed by the filter-aided sample preparation (FASP) procedure as previously described with minor modifications [28,29]. Briefly, 200 µg of proteins from each sample were mixed with 30 μL SDT buffer (4% SDS, 150 mM Tris-HCl, 100 mM DTT, pH 8.0). The mixed suspension was then loaded into centrifugal ultrafiltration units (Microcon units, 10 kD) and centrifuged to remove the detergent, dithiothreitol (DTT), and other low-molecular-weight components with UA buffer (8 M Urea, 150 mM Tris-HCl, pH 8.0). To block the reduced cysteine residues, 100 µL of iodoacetamide (100 mM iodoacetamide in UA buffer) was added, and the samples were incubated for 30 min in darkness. After centrifugation, the filters were washed three times with 100 µL UA buffer, and then other two times with 100 µL of 25 mM NH_4_HCO_3_ buffer. Finally, the proteins were dissolved in 40 μL 25 mM NH_4_HCO_3_ buffer (containing 4 μg trypsin) and incubated overnight at 37 °C. After centrifugation, the peptides were collected and followed by desalting treatment using C18 cartridges (Empore™ SPE Cartridges C18, standard density, Sigma).

For LC-MS/MS analysis, the Q Exactive mass spectrometer that coupled to Easy nLC (Thermo Fisher Scientific, Waltham, MA, USA), was applied. The peptide mixture of above was placed into the C18-reversed phase analytical column (Thermo Fisher Scientific, USA), which was packed with buffer A (0.1% Formic acid), and separated with a linear gradient of buffer B (84% acetonitrile and 0.1% Formic acid) at a flow rate of 300 nl/min controlled by IntelliFlow technology. The positive ion mode was used in a mass spectrometer. MS date was obtained by data-dependent top 10 method dynamically selecting the most abundant precursor ions from the survey scan (300–1800 m/z) in order to acquire the higher energy collisional dissociation (HCD) fragmentation. The instrument was running with peptide recognition mode enabled. The survey scans were performed under the conditions of 70,000 resolution at 200 m/z, while the HCD spectra were the 17,500 at 200 m/z. Dynamic exclusion duration was 40.0 s. Normalized collision energy was set to 30 eV, and the underfill ratio was defined as 0.1%.

For the identification and quantification of proteins, Maxquant Software version 1.5.3.17 [30] was applied, which combined and searched the MS raw data with the database. The proteins were searched against the Uniprot *Paralichthys olivaceys* protein database. The max missed cleavages of trypsin enzyme were set as 2, while the carbamidomethylation of cysteine residues and oxidation of methionine were, respectively, set as fixed and variable modifications. For MS scans, the mass error tolerances of the first search and main search were, respectively, set as 20 ppm and 6 ppm. For MS/MS scans, the mass tolerance was set as 20 ppm. Label-free quantification (LFQ) was chosen for protein quantification and the LFQ min ratio count was set as 1. The LFQ intensity is often used in the comparison between groups and reveals the level of relative protein expression [31]. The false discovery rate (FDR) of peptide and protein levels was set as 0.01 to filter the results.

### 2.5. Identification of Differentially Abundant Proteins (DAPs)

To identify differentially abundant proteins (DAPs), a two-sided Student’s *t*-test was applied for each protein comparison between groups, generating fold changes and *p*-values. Fold change was calculated as the ratio of the average protein expression in the experimental group versus the control group. The *p*-value was then adjusted for multiple hypothesis testing using the Benjamini–Hochberg false discovery rate correction. Proteins meeting the dual criteria of adjusted *p*-value < 0.05 and |fold change| > 1.5 were regarded as DAPs and considered statistically and biologically significant. This threshold has been commonly adopted in proteomic studies to identify DAPs [32,33]. Moreover, proteins that were detected in one group but entirely absent in the other group were also considered to be DAPs with substantial differences. The total number of identified DAPs is summarized in the histogram.

### 2.6. GO and KEGG Annotation Analysis

For the GO annotation of DAPs, the NCBI BLAST+ client software (version 2.13.0+) was used for locally searching the protein sequences of DAPs. The InterProScan (version 5.59-91.0) was adopted to find homolog sequences, and GO terms were then mapped. Protein sequences were annotated using the Blast2GO software (version 6.0.3) program, and the annotated GO results were plotted by R scripts (version 4.2.1). For KEGG annotation of DAPs, DAPs were blasted using the KEGG database (http://www.genome.jp/kegg/) (accessed on 16 June 2024) to retrieve KEGG orthology identification and then subsequently mapped to pathways in KEGG.

### 2.7. Construction of the Interaction Network and Key Proteins Selection

The DAPs of the LI-LC group and the HI-HC group were, respectively, used to construct protein–protein interaction (PPI) networks using the String software (version 12.0) (http://string-db.org/) (accessed on 18 June 2024) as reported previously [34]. The results obtained from String software were then imported into Cytoscape software (version 3.9.1) (http://www.cytoscape.org/) (accessed on 18 June 2024) to visualize and further analyze functional PPI networks. The PPI degree of each protein was calculated to evaluate its importance in the PPI networks. The key proteins were selected from PPI networks based on the PPI ≥ 6 and |fold change| ≥ 2.

### 2.8. Key Proteins Analysis and Validation

A Venn diagram was employed to analyze the unique key proteins present in both groups. The blue and pink circles represent the HI-HC and LI-LC groups, respectively, with the numerical values inside each circle indicating the corresponding number of key proteins. To validate the proteomics data, nine key proteins were randomly selected and quantified using quantitative real-time PCR (qRT-PCR) via the comparative threshold cycle (2^−ΔΔCt^) method. α-Tubulin (TUBA) was used as the reference gene for data normalization [35,36]. The primers were designed using NCBI Primer-BLAST (https://www.ncbi.nlm.nih.gov/tools/primer-blast/) (accessed on 10 September 2024) and listed in Table 1.

## 3. Results

### 3.1. A Large Number of Proteins Exhibit Differential Expressions in Flounder Infected with E. tarda at Low and High Temperatures

**Figure 1:**
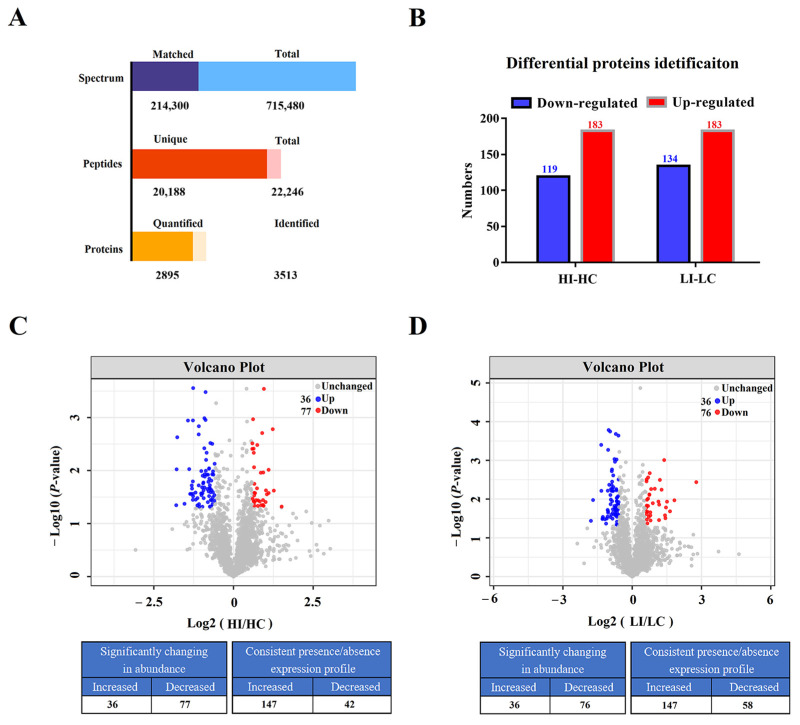
Summary of the proteomic results. (**A**) Statistics of proteomic sequencing and annotation. Spectra: the number of mass spectra after quality control; unique peptide: a specific peptide in a group of proteins. (**B**) The histogram displays the number of DAPs in the HI-HC group and the LI-LC group. (**C**) Volcano plot visualizing the distribution of DAPs in the HI-HC group. (**D**) Volcano plot visualizing the distribution of DAPs in the LI-LC group.

In this study, flounder were infected with *E. tarda* under low temperature of 15 °C (LI) or high temperature of 23 °C (HI), and the uninfected fish were similarly maintained at low (LC) or high (HC) temperature. Label-free proteomics was performed to compare the protein expression profiles in the spleen of the fish. A total of 715,480 spectrums (214,300 matched), 22,246 peptides (20,188 unique), and 3513 proteins (2895 quantified) were identified (Figure 1A). The molecular weight, peptide counts, peptide length, and peptide coverage distribution of the 3513 proteins are displayed in Figure S1. For differentially abundant proteins (DAPs), a total of 302 DAPs in the HI-HC group were identified, with 183 DAPs upregulated and 119 DAPs downregulated (Figure 1B,C; Table S1). In the LI-LC group, a total of 317 DAPs in LI-LC group were identified, with 183 DAPs upregulated and 134 DAPs downregulated (Figure 1B,D; Table S2).

### 3.2. GO and KEGG Analyses

**Figure 2:**
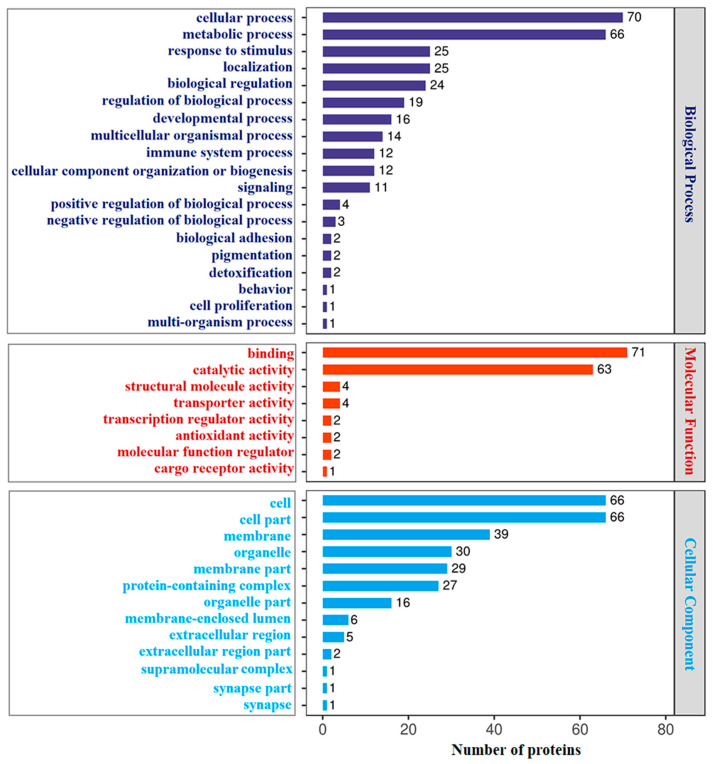
GO analysis of the DAPs in the HI-HC group. The top 20 GO terms (level 2) in the categories of biological process (dark blue), molecular function (red), and cellular component (bright blue). The *Y*-axis represents the protein functional classification, and the *X*-axis represents the corresponding number of DAPs.

**Figure 3:**
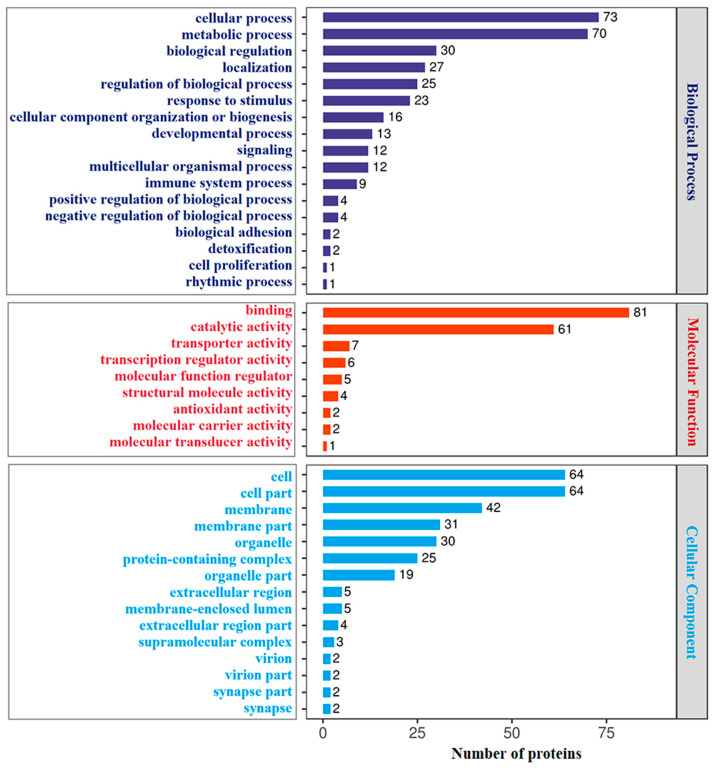
GO analysis of the DAPs in the LI-LC group. The top 20 GO terms (level 2) in the categories of biological process (dark blue), molecular function (red), and cellular component (bright blue). The *Y*-axis represents the protein functional classification, and the *X*-axis represents the corresponding number of DAPs.

GO annotation showed that 302 DAPs identified in HI-HC group were classified into three main categories: biological process (BP), molecular function (MF) and cellular component (CC). The top 20 GO terms of each category are shown in Figure 2. In the BP category, cellular process, metabolic process, and response to stimulus were the top three terms. Other terms included immune system process, signaling, biological adhesion and cell proliferation. In the MF category, binding, catalytic activity, and structural molecule activity were the top three terms. Other terms include transcription regulator activity and antioxidant activity. In the category of CC, cell, cell part and membrane were the top three annotated terms. Similarly, among the 317 DAPs identified in the LI-LC group, the top 20 annotated GO terms are shown in Figure 3. In the BP category, cellular process, metabolic process, and biological regulation were the top three terms. Other terms included response to stimulus, signaling, immune system process, biological adhesion, and cell proliferation. In the MF category, binding, catalytic activity, and transporter activity were the top three terms. Other terms include transcription regulator activity and antioxidant activity. In the category of CC, cell, cell part, and membrane were the top three annotated terms. Comparatively, in the BP category, cellular process and metabolic process were the two most enriched terms in both groups. Meanwhile, response to stimulus ranked third in the HI-HC group, and biological regulation was the third most enriched term in the LI-LC group. Within the MF category, binding and catalytic activity were also the top two terms in both groups, followed by structural molecule activity in the HI-HC group and transporter activity in the LI-LC group in third place. For the CC category, the top three terms were consistent across both groups.

**Figure 4:**
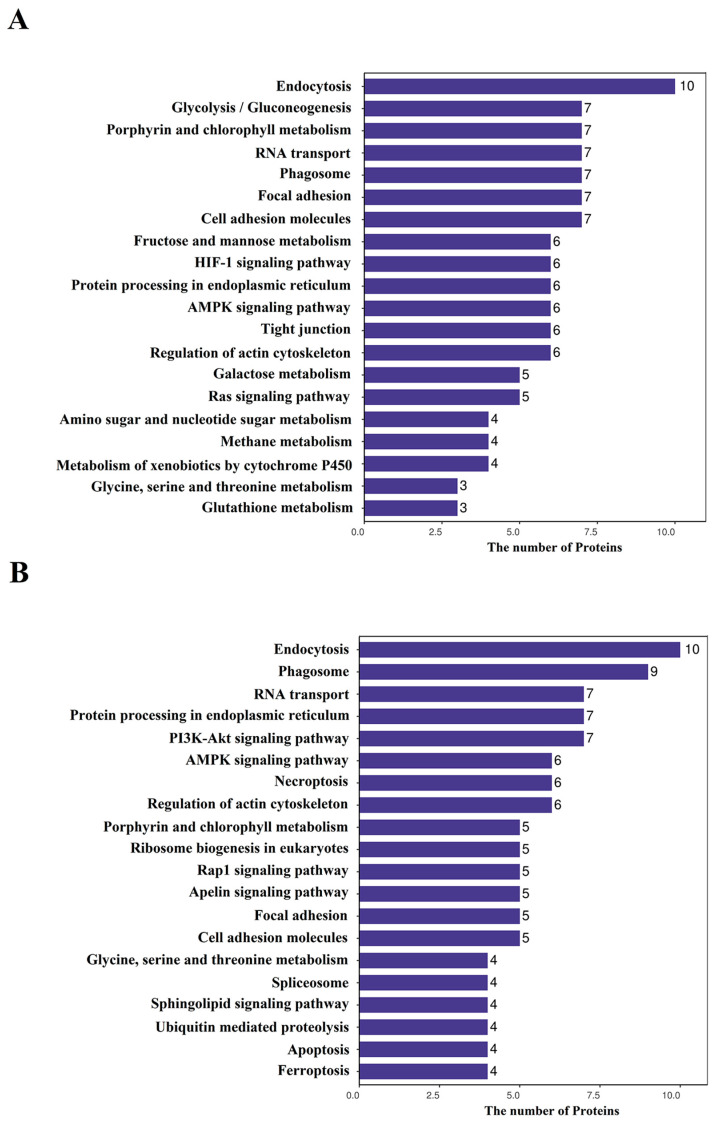
The top 20 KEGG pathways of DAPs in the HI-HC group (**A**) and the LI-LC group (**B**). The *X*- and *Y*-axis represent the DAP number and the KEGG pathway, respectively.

KEGG analysis of the DAPs in the HI-HC group revealed that the top 20 KEGG pathways included seven immune-related pathways, i.e., Endocytosis, Phagosome, Focal adhesion, Cell adhesion molecules, HIF-1 signaling pathway, AMPK signaling pathway, and Ras signaling pathway (Figure 4A). In the LI-LC group, twelve immune-related pathways were enriched, including the P13K-Akt signaling pathway, the AMPK signaling pathway, Endocytosis, Phagosome, Necroptosis, Rap1 signaling pathway, Apelin signaling pathway, Focal adhesion, Cell adhesion molecules, Sphingolipid signaling pathway, Apoptosis, and Ferroptosis (Figure 4B). Comparatively, the AMPK signaling pathway, Phagosome, Focal adhesion, Endocytosis, and Cell adhesion molecules were commonly enriched pathways in both groups. Especially, the HIF-1 signaling pathway and Ras signaling pathway were exclusively enriched in the HI-HC group, whereas the P13K-Akt signaling pathway, Necroptosis, Rap1 signaling pathway, Apelin signaling pathway, Sphingolipid signaling pathway, and Apoptosis and Ferroptosis were unique to the LI-LC group.

### 3.3. Construction of Interaction Network and Key Proteins Identification

**Figure 5:**
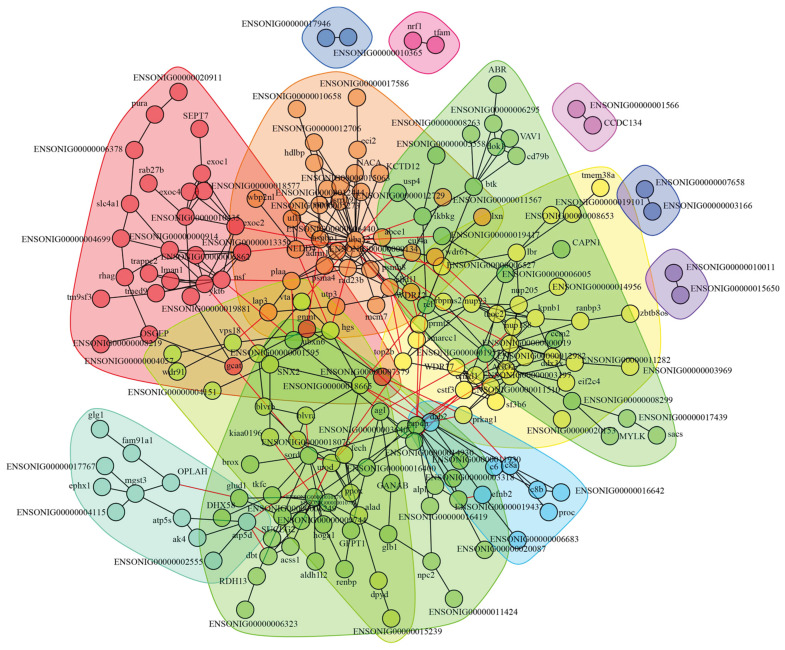
Protein–protein interaction (PPI) networks of DAPs in the HI-HC group using the STRING online platform and Cytoscape software. In this network, Nodes represented the DAPs. Lines between the nodes represented the protein interaction relationships. The interaction networks of proteins belonging to the same class were clustered together with the same background color. Different background colors represented different classes of protein interaction network.

**Figure 6:**
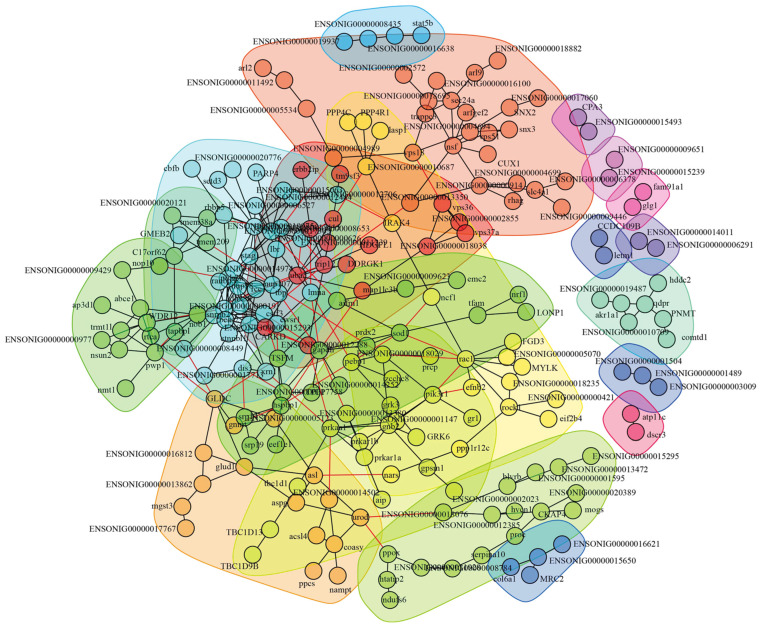
Protein–protein interaction (PPI) networks of DAPs in the LI-LC group using the STRING online platform and Cytoscape software. In this network, Nodes represented the DAPs. Lines between the nodes represented the protein interaction relationships. The interaction networks of proteins belonging to the same class were clustered together with the same background color. Different background colors represented different classes of the protein interaction network.

The identified 302 DAPs in the HI-HC group and 317 DAPs in the LI-LC group were, respectively, used to construct the protein–protein interaction (PPI) network. In the HI-HC group, 197 DAPs turned out to exhibit interactions with each other and form PPI networks (Figure 5). The 197 DAPs with interactions were clustered into 13 classes, and DAPs of the same class were clustered together with the same background color. To identify key proteins in PPI networks, the threshold of PPI degree ≥ 6 and |fold change| ≥ 2 was further set. As a result, a total of 27 key proteins were identified, with 20 key proteins upregulated and 7 key proteins downregulated (Table 2). In the LI-LC group, 213 DAPs exhibited interactions with each other and formed PPI networks (Figure 6). The 213 DAPs with interactions were clustered into 20 classes, and a total of 26 key proteins were identified, with 16 key proteins upregulated and 10 key proteins downregulated (Table 2).

### 3.4. Special Key Proteins Analysis in HI-HC Group

**Figure 7:**
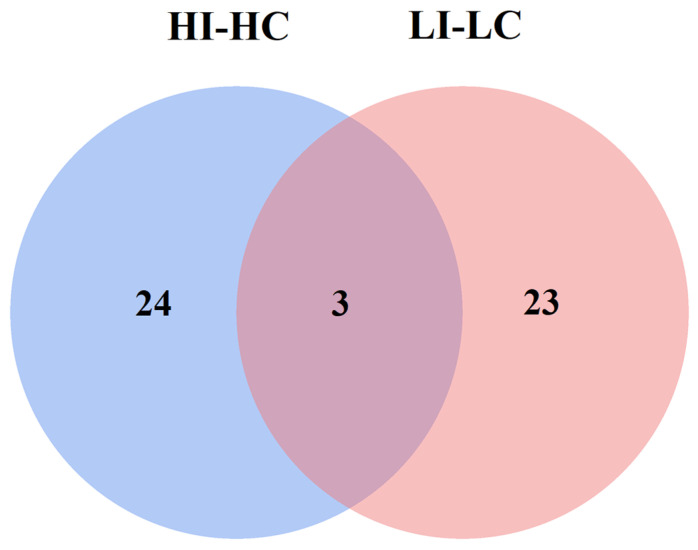
Venn diagram analysis of the key proteins between the HI-HC group and the LI-LC group. The blue circle represents the HI-HC group. The pink circle represents the LI-LC group. The value inside the circle represents the number of key proteins.

According to the Venn diagram analysis results displayed in Figure 7, twenty-four key proteins were identified specifically in the HI-HC group. Out of these, eight key immune-related proteins were further identified, which were mainly involved in the endocytosis process, as demonstrated in Table 3. More specifically, four key proteins, namely YKT6, HGS, TFRC, and RAB11B, that are closely associated with the endocytosis process were markedly upregulated, while the C8A of the complement membrane attack complex was also hugely upregulated. Apart from these, the other three immune-related proteins, namely ELAVL1, ROCK2, and EXOC2, exhibited substantial upregulation. Taken together, these findings suggest that infection of flounder by *E. tarda* under high temperature leads to enhanced cell endocytosis capacity as well as increased complement activity.

### 3.5. Special Key Proteins Analysis in LI-LC Group

Based on the results of Venn diagram analysis presented in Figure 7, twenty-three key proteins were identified specifically in the LI-LC group. Further analysis revealed the identification of nine key immune-related proteins, which were primarily involved in histone and RNA transport processes as shown in Table 3. Specifically, two key proteins belonging to the histone, namely H3 and macroH2A1, exhibited hugely elevated levels. On the other hand, three nuclear pore proteins (NUP107, NUP214, and NUP98) that are closely associated with RNA transport were markedly impacted with varying patterns. NUP107 and NUP214 were hugely downregulated, while NUP98 was greatly upregulated. Additionally, four other immune-related proteins (RAC1, POLR2H, PRKAA1, and PIK3R1) were also affected with different trends. Overall, these findings suggest that infection of flounder by *E. tarda* under low temperature leads to increased histone abundance and interferes with the normal RNA transport of the host.

### 3.6. Shared Key Proteins Analysis Between HI-HC and LI-LC Groups

Based on the Venn diagram analysis results shown in Figure 7 and Table 3, three proteins were shared among both groups including NSF, CSTF3, and XPO1. Among these, both NSF and CSTF3 showed similar up-regulation trends and comparable PPI degrees under high and low temperature conditions, suggesting that their expression may not be significantly affected by temperature change and is more likely associated with *E. tarda* infection. Notably, XPO1 was strongly down-regulated in the HI-HC group but markedly up-regulated in the LI-LC group, indicating that its expression is highly sensitive to temperature variation.

### 3.7. Key Proteins Validation

**Figure 8:**
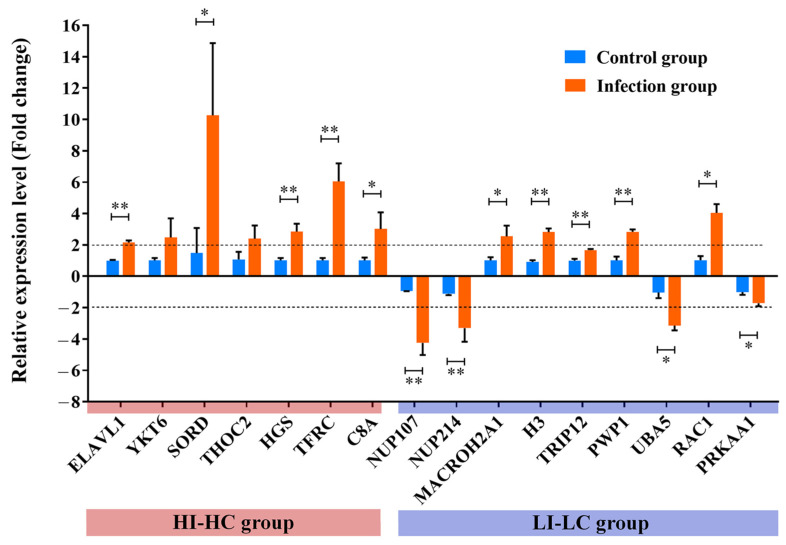
Validation of key proteins by qRT-PCR. To confirm the express level of the differentially expressed genes in proteomics, sixteen key proteins were randomly selected and determined by qRT-PCR using the comparative threshold cycle (2^−ΔΔCt^) method. α-tubulin was used to normalize data. The expression level in the control group was normalized to 1. The *X*-axis indicates the genes examined. The *Y*-axis represents the relative expression level (fold change), reflecting normalized gene expression in the infection group relative to the control group. Data are expressed as the mean ± SEM (N = 3). Data were analyzed with Student’s *t*-test, and statistical significance was defined as *p* < 0.05. *, *p* < 0.05; **, *p* < 0.01. The dotted line represents the cut-off for a 2-fold change.

The validation of key proteins was carried out by detecting their mRNA levels using qRT-PCR. The results, as presented in Figure 8, revealed that the regulation trends of the sixteen randomly selected key proteins in transcript levels were consistent with the trends observed in the proteomic data shown in Table 2, which further confirms the reliability of the proteomic data.

## 4. Discussion

The outbreak of fish diseases not only depends on the bacteria’s pathogenicity but also on the environmental conditions, including temperature changes. *E. tarda*, a known pathogen to flounders, has become one of the most serious threats to flounder farming [18,19]. The specific challenges posed by *E. tarda* to flounder farming are multifaceted and severe. Firstly, this pathogen induces extremely high mortality rates. Edwardsiellosis outbreak typically results in cumulative mortality of 50–80% in susceptible fish populations within a short period [37]. Secondly, the economic impact is substantial. Although precise statistical data are limited, the high mortality rate clearly results in significant economic losses, enabling *E. tarda* as one of the most serious pathogens threatening the economic sustainability of flounder aquaculture. Finally, infections caused by *E. tarda* occur predominantly in summer and autumn, with the highest prevalence observed in July and August when water temperatures are elevated, representing the peak occurrence period [38]. Additionally, *E. tarda* can persist in asymptomatic carrier fish, thus facilitating silent transmission and triggering sudden disease outbreaks under stressful conditions. In our previous study, we found that high temperature enhanced *E. tarda* dissemination in flounder tissues compared to low temperature, and the metabolic profile induced by high temperature was characterized by extensively decreased amino acids [39]. However, the underlying mechanism has not been fully explained, especially the changes in protein level. In this study, we adopted the label-free proteomics to further reveal the protein expression profiles of flounder exposed to *E. tarda* infection under different temperatures (15 °C, 23 °C) stimulation. A large number of proteins in response to *E. tarda* infection under two temperatures were identified and displayed huge differences. In detail, the endocytosis capacity and complement activity were especially enhanced in the HI-HC group, while the histone abundance and the normal RNA transport of the host in the LI-LC group were, respectively, upregulated and interfered.

Endocytosis is a process utilized by phagocytes to internalize bacteria, which are then targeted and destroyed in the lysosome [40]. In this study, four key immune-related proteins (YKT6, HGS, TFRC, RAB11B) were closely involved in the endocytosis process and upregulated with a huge difference in response to *E. tarda* infection under high temperature, indicating that the endocytosis capacity of immune cells toward *E. tarda* was enhanced. Among these proteins, YKT6 (synaptobrevin homolog YKT6) is a recognition molecule for SNARE (soluble NSF attachment protein receptor) and has been reported to facilitate endocytosis [41,42]. In *Drosophila melanogaster*, YKT6 was identified as an essential regulator of parasite phagocytosis, and knockdown of YKT6 led to a significant reduction in parasite phagocytosis by drosophila S2 cells [41]. Similarly, YKT6 in mammalian cells was found to exist in peripheral vesicles and be associated with the endocytic pathway [42]. HGS (hepatocyte growth factor-regulated tyrosine kinase substrate) is an early endosomal protein and plays a role in regulating the trafficking of cargos from the early endosome to the late endosome [43,44]. In mammals, HGS in mice exhibited defective endocytic function of the endosome, and the overexpression of HGS influenced the morphology of the early endosome [43]. TFRC (transferrin receptor protein 1) plays an important role in cell immune activities and cellular iron uptake via the receptor-mediated endocytosis [45]. In fish, the expressions of TFRC in Nile tilapia macrophages/monocytes were significantly upregulated upon *Streptococcus agalactiae* stimulation, and its ligand, transferrin, also promoted monocyte/macrophage phagocytosis [46,47]. RAB11B (Ras-related protein Rab11B) is a critical regulator of membrane trafficking of endocytic pathways, and the deletion of RAB11B in the human HeLa cell line resulted in the enlargement of early endosomes and interfered with the endosomal-lysosomal pathway [48]. Taken together, these results indicated that the endocytosis capacity of flounder immune cells is enhanced in response to *E. tarda* infection under elevated temperature conditions.

Similar trends have been reported in other fish species, such as rainbow trout, sablefish, and cyprinid fish [8,49,50]. For instance, in rainbow trout, blood phagocytes acclimatized to 20 °C exhibited enhanced phagocytic and complement activity compared to those at 5 °C [8]. Similarly, in sablefish (*Anoplopoma fimbria*), kidney immune cells demonstrated significantly elevated phagocytic capacity at 18 °C relative to 8 °C [49]. In cyprinid fish (*Tinca tinca* L.), blood granulocytes also showed improved phagocytosis of *Candida albicans* at 30 °C compared to 22 °C [50]. However, contrasting results have been observed in other species. A study in tilapia (*Oreochromis mossambicus*) reported a significant reduction in the phagocytic capacity of blood leukocytes toward formalin-killed *Escherichia coli* when temperatures were shifted from 27 °C to either lower (19 °C or 23 °C) or higher (31 °C or 35 °C) temperatures [6]. In common carp (*Cyprinus carpio*), pronephric macrophages exhibited the highest phagocytic index against yeast (*Saccharomyces cerevisiae*) at 12 °C compared to 20 °C or 28 °C [51]. These discrepancies suggest that the effect of temperature on phagocytic capacity varies across fish species and may be influenced by thermal adaptation ranges, the specific immune cell types examined, and the nature of the antigen involved. Such factors may help explain the divergent responses observed in tilapia and common carp.

Complement is a humoral factor of innate immunity and plays a crucial role in the fish immune response to pathogen infection, including C1q, C3, C5b, C6, C7, C8, C9, etc. [52]. Among these, C5b recruits C6, C7, C8, and C9 to form the MAC (membrane attack complex), leading to the cytolysis of the lipid membrane of pathogens [53]. Previous study in carp indicated that C8 played an indispensable role in the hemolytic lysis of rabbit red blood cells [54]. In this study, we discovered that C8A (C8 alpha chain) was hugely upregulated under high temperature, suggesting that the complement activity of flounder was enhanced and likely caused MAC formation that ultimately led to the lysis of *E. tarda*. Similar results were observed in tilapia, where the plasma alternative complement pathway (ACH_50_) significantly increased as the water temperature rose from 27 °C to high temperatures (31 °C and 35 °C) [6]. In gilthead seabream (*Sparus aurata*), the lowest serum complement activity was recorded in the coldest month (January), and the highest serum complement activity was recorded under the hottest season (autumn) [55]. Therefore, temperature changes have a significant impact on fish complement activity. The hugely upregulated expression of C8A in our study suggests that the complement activity of flounder is enhanced under high temperature in response to *E. tarda* infection.

Histones play a crucial role not only in the packaging of chromosomal DNA, but also in host immune responses against pathogens. Accumulating evidence has demonstrated that histones exhibit broad-spectrum antimicrobial activity across species. In fish, this function has been directly demonstrated: histone H2B and H4 from half-smooth tongue sole (*Cynoglossus semilaevis*) bind and inhibit both Gram-negative and Gram-positive bacteria, including *Pseudomonas fluorescens* (*P. fluorescens*), *Vibrio anguillarum* (*V. anguillarum*), *Staphylococcus aureus* (*S. aureus*), and *Micrococcus luteus* (*M. luteus*) [56]. Most importantly, histone H3.3 in flounder exhibits broad bacterial-binding capacity and inhibits the growth of multiple pathogens, including *P. fluorescens*, *V. anguillarum*, and *M. luteus* [57]. These findings are supported by studies in mammals, where histones (H2A, H2B, and H4) derived from neutrophil extracellular traps or other cells show activity against bacteria like *S. aureus* and *Shigella flexneri* [58,59] and parasites like *Leishmania amazonensis* and *Leishmania major* [60], highlighting an evolutionarily conserved mechanism. In this study, we observed significant upregulation of histone H3 and macroH2A1 in flounder under low temperature (15 °C), suggesting a crucial role of these histones in the immune response to *E. tarda* infection under cold conditions. This is consistent with the established role of flounder H3.3 in enhancing resistance to *E. tarda* and upregulating immune-related genes such as *IFN-γ* and *MHC-Iα* [57]. While the antimicrobial function of H3.3 is thus partially established in flounder, the role of macroH2A1 in antibacterial immunity remains unclear and warrants further investigation.

Gene transcription and translation depend heavily on the efficient transport of biological macromolecules, a process facilitated by the nuclear pore complex (NPC) [61,62]. Composed predominantly of nucleoporins (Nups), the NPC regulates nucleocytoplasmic transport and thereby influences the expression and translation of immune-related genes [61]. In this study, three Nups (NUP214, NUP98, and NUP107) in the LI-LC group exhibited distinct regulatory patterns: NUP214 and NUP107 were downregulated, whereas NUP98 was upregulated. In mammals, NUP98 is critical for RNA export and exhibits transcription-dependent mobility within cells [63]. In humans, NUP98 fusion proteins have been reported to inhibit the CRM1-mediated nuclear export of transcription factors and lead to the leukemogenic disease [63]. Nup214 serves as a terminal binding site in nuclear protein export, forms a subcomplex with Nup88, and interacts with CRM1 to regulate cargo molecules transport [64]. A previous study in human Hela cells indicated that depletion of Nup214 by RNA interference (RNAi) strongly reduced CRM1-mediated export of the transcription factor, underscoring the important role of Nup214 in CRM1-mediated nuclear protein export in vivo [64]. NUP107, another essential component, is required for the structural integrity of the NPC and the proper assembly of other Nups [65]. Its knockdown in HeLa cells not only disrupted NPC composition but also partially inhibited mRNA export [65]. Furthermore, in human cardiac myocytes, NUP107 modulates the nucleocytoplasmic trafficking of Scn5a mRNA, thereby regulating cardiac electrical activity [66]. Despite their well-characterized roles in mammals, the functions of NUP214, NUP98, and NUP107 in fish remain poorly understood. The complex regulation of these Nups in flounder under *E. tarda* infection suggests that low temperature significantly interferes with nuclear mRNA export, ultimately impairing the transcription and translation of immune genes.

Based on the proteomic analysis, three shared key proteins (NSF, CSTF3, and XPO1) exhibit distinct functional roles and expression patterns. NSF (Vesicle-fusing ATPase) is involved in intracellular membrane fusion processes, particularly in vesicular trafficking [67]. Its consistent up-regulation under both high and low temperatures suggests its role is primarily associated with the infection response rather than temperature variation. Given that the study found high temperature promoted endocytosis, the function of NSF in mediating vesicle fusion could be critical for the processes leading to pathogen internalization. CSTF3 (Cleavage stimulation factor subunit 3) plays a key role in mRNA polyadenylation and maturation [68]. Like NSF, it showed stable up-regulation across temperatures, indicating its importance in post-transcriptional regulation during immune challenge. The immune response requires the rapid production of numerous immune-related proteins, and CSTF3 is fundamental for enabling the expression of these genes. In contrast, XPO1 (Exportin-1), which mediates nuclear export of proteins and RNAs, displayed strong temperature sensitivity [69]. It was significantly down-regulated at high temperature (23 °C) but up-regulated at low temperature (15 °C). This suggests that function of XPO1 in nucleocytoplasmic transport is highly influenced by temperature, potentially contributing to altered immune gene expression and RNA transport efficiency under different thermal conditions.

Of note, our previous study has indicated that high temperature enhanced the dissemination of *E. tarda* in flounder tissues compared to lower temperature, suggesting that *E. tarda* evaded the kill of host [39]. However, in this study, high temperature was found to enhance both phagocytic activity and complement function. Given that complement activation also facilitates phagocytosis, these results suggest increased internalization of *E. tarda* into host cells, which would typically be expected to promote bacterial clearance. Nevertheless, *E. tarda* can employ multiple mechanisms to evade host immune killing including resistance to serum killing [70] and inhibition of apoptosis [71]. Previous studies have also demonstrated its ability to escape from endocytic vesicles and replicate within the host cytoplasm [72]. It is therefore possible that in the present study, *E. tarda* utilizes specific virulence factors to avoid phagocytic killing, thereby promoting bacterial dissemination.

Based on our findings, we propose the following recommendations for flounder aquaculture to combat *E. tarda* outbreaks. Firstly, enhanced monitoring should be implemented during high-temperature periods, particularly in summer and autumn (July–August), including the adoption of cooling measures such as increasing water exchange or utilizing deeper groundwater to mitigate the risk of severe outbreaks. Furthermore, given the crucial role of the histone-mediated immune response at lower temperatures, strategic breeding programs focusing on selecting broodstock with enhanced expression of antimicrobial histones (e.g., H3) could be promising for developing flounder lineages with broad-spectrum resistance across varying temperatures. Additionally, considering that elevated temperatures enhance endocytosis and complement activity, administering vaccines prior to the high-temperature season may elicit a more robust and protective immune response.

## 5. Conclusions

In this study, we employed label-free proteomics to analyze the immune response of flounder to *E. tarda* infection under low and high temperatures. A total of 317 and 302 DAPs were identified in the LI-LC and HI-HC groups, respectively. GO and KEGG analyses revealed numerous immune-related proteins and pathways in both groups. Among these, twenty-six key DAPs in the LI-LC group and twenty-seven key DAPs in the HI-HC group were identified and found to form extensive interaction networks. Further Venn analysis screened out uniquely expressed immune-related DAPs in each group, which were discussed in detail. The results demonstrate that endocytic capacity and complement activity were enhanced under high temperature, whereas histone expression was upregulated and RNA transport was significantly disrupted under low temperature. Our findings highlight a clear divergence in the immune response of flounder to *E*. *tarda* infection between 15 °C and 23 °C, providing valuable insights into how temperature variation influences antibacterial immunity in fish.

## Figures and Tables

**Table 1:** List of primers used for qRT-PCR.

Gene Name	Gene ID	Forward Primer (5′-3′)	Reverse Primer (5′-3′)
ELAVL1	XM_020107316.1	GATGTTTGGGCCGTTTGGTG	CCCAGTCGATACCCGTTCAG
YKT6	XM_020099185.1	ATCTCGTGGCAAAGTCGGAA	AGCAGAAAGGCAGAGACGAG
SORD	XM_020080198.1	TTGGCAGATTGGTAGTGAGTCT	AACCCGATAGGCCCTGAAGA
THOC2	XM_020085721.1	AATGAGGAAACCCCCACGTC	CACAACCATGACCAGCTTGC
HGS	XM_020094525.1	GGCCCGTTACCTGAACAGAA	ACTGCTGCTCCACTATGCTG
TFRC	XM_020105708.1	GCAGCTCTGTCCAACCATCT	ACTGGCTGTCGGTGAATTGT
C8A	XM_020099517.2	ACGTGCAAGTGGGACTCAAA	GCGGGAGTACATCCCAAAGT
NUP107	XM_020112821.1	ATCGACTGGCTGCTGTTTGA	TCATCGAGTCCTCCGGAACT
NUP214	XM_020081961.1	AGGGTCTTTTCACAACACTGG	TCACTGGTGAGATCTTGACTG
H3	XM_020109331.1	GTGGTTCAGTTGTGCGTCC	CAGGTTGGCGTCTGAGAAC
macroH2A1	XM_020086110.1	CCCGACCAACTCCTCCATCTA	TGTGGCAGTGAATGACGTACT
TRIP12	XM_020095042.1	GCCCCTCAGCTGATGTGAAA	TGGAATTAGGCCGGTTGGAC
PWP1	XM_020092036.1	TCTGCTGCCAGCTTATCCAC	AGGAGTCATGTTGCCCACAG
UBA5	XM_020087372.1	CTCCAACCCGTACAGTCGTC	AGCAGCTTACCAATGCCACA
RAC1	XM_020104358.1	TGGGGCTAAGTAAACGCTGT	GGTGCAGCCTCAACAATCAGT
PRKAA1	XM_020082451.1	CAAAAGTCCACGTCCCACCT	CAAAAGTGCTTCCTGCGTCC
α-Tubulin	XM_020111916.1	TGACATCACAAACGCCTGCTTC	GCACCACATCTCCACGGTACAG

ELAVL1: ELAV-like protein 1; YKT6: Synaptobrevin homolog YKT6; SORD: Sorbitol dehydrogenase; THOC2: THO complex subunit 2; macroH2A1: Core histone macro-H2A.1; TRIP12: E3 ubiquitin-protein ligase TRIP12; PWP1: Periodic tryptophan protein 1; UBA5: Ubiquitin-like modifier-activating enzyme 5; PRKAA1: 5′-AMP-activated protein kinase catalytic subunit alpha-1.

**Table 2:** List of the key proteins based on the threshold of protein–protein interaction (PPI) degree ≥ 6 and |fold change| ≥ 2 in HI-HC group (left) and LI-LC group (right). The symbol “−∞” indicates that the protein was detected in the HC group/LC group but not detected in the HI group/LI group. Conversely, “+∞” indicates that the protein was detected in the HI group/LI group but not detected in the HC group/LC group.

HI-HC Group						LI-LC Group					
Protein ID	Name	PPI Degree	Fold Change	*p*-Value	Regulation	Protein ID	Name	PPI Degree	Fold Change	*p*-Value	Regulation
XP_019954006.1	UBA52	32	+∞	NA	Up	XP_019964890.1	H3	12	+∞	NA	Up
XP_019953060.1	ALDH18A1	11	+∞	NA	Up	XP_019966822.1	XPO1	11	+∞	NA	Up
XP_019938172.1	RAD23B	11	−2.12	1.46 × 10^−3^	Down	XP_019941669.1	macroH2A1	11	+∞	NA	Up
XP_019962875.1	ELAVL1	10	+∞	NA	Up	XP_019968380.1	NUP107	11	−∞	NA	Down
XP_019954477.1	CSTF3	10	+∞	NA	Up	XP_019950601.1	TRIP12	10	+∞	NA	Up
XP_019935757.1	SORD	10	+∞	NA	Up	XP_019937520.1	NUP214	10	−∞	NA	Down
XP_019966822.1	XPO1	9	−∞	NA	Down	XP_019959917.1	RAC1	10	+∞	NA	Up
XP_019954744.1	YKT6	9	+∞	NA	Up	XP_019954477.1	CSTF3	9	+∞	NA	Up
XP_019933706.1	NSF	9	+∞	NA	Up	XP_019954372.1	DHX38	9	+∞	NA	Up
XP_019950084.1	HGS	8	+∞	NA	Up	XP_019947595.1	PWP1	9	+∞	NA	Up
XP_019935975.1	CHD1L	8	−∞	NA	Down	XP_019954034.1	POLR2H	9	−2.06	2.10 × 10^−2^	Down
XP_019941280.1	THOC2	8	+2.07	2.81 × 10^−2^	Up	XP_019969351.1	WDR12	9	−∞	NA	Down
XP_019964808.1	AGL	7	+∞	NA	Up	XP_019942931.1	UBA5	8	−∞	NA	Down
XP_019958157.1	EXOC2	7	+∞	NA	Up	XP_019938010.1	PRKAA1	8	−∞	NA	Down
XP_019969379.1	KPNB1	7	−2.28	3.54 × 10^−2^	Down	XP_019933706.1	NSF	8	+∞	NA	Up
XP_019940542.1	ROCK2	7	+∞	NA	Up	XP_019966497.1	NUP98	7	+∞	NA	Up
XP_019961267.1	TFRC	7	+∞	NA	Up	XP_019963564.1	CTNNBL1	7	+∞	NA	Up
XP_019936097.1	ATP5D	6	+∞	NA	Up	XP_019954792.1	GLDC	7	+2.84	1.20 × 10^−2^	Up
XP_019935808.1	CUL4A	6	−∞	NA	Down	XP_019957398.1	SUN1	7	−∞	NA	Down
XP_019963351.1	ALDH3B1	6	+∞	NA	Up	XP_019953698.1	STAG1	6	+∞	NA	Up
XP_019937551.1	NUP188	6	−∞	NA	Down	XP_019968563.1	NOB1	6	−∞	NA	Down
XP_019946175.1	TOP2B	6	+∞	NA	Up	XP_019937802.1	PIK3R1	6	+∞	NA	Up
XP_019965190.1	RAB11B	6	+∞	NA	Up	XP_019941399.1	SEC24A	6	+∞	NA	Up
XP_019951363.1	PLAA	6	−∞	NA	Down	XP_019965029.1	TSFM	6	−∞	NA	Down
XP_019955076.1	C8A	6	+∞	NA	Up	XP_019961505.1	HSPBP1	6	+∞	NA	Up
XP_019964134.1	CRNKL1	6	+∞	NA	Up	XP_019966512.1	CUL1	6	−∞	NA	Down
XP_019965019.1	GCK	6	+2.03	2.37 × 10^−2^	Up						

UBA52: Ubiquitin-ribosomal protein eL40 fusion protein; ALDH18A1: Delta-1-pyrroline-5-carboxylate synthase; RAD23B: UV excision repair protein RAD23 homolog B; ELAVL1: ELAV-like protein 1; CSTF3: Cleavage stimulation factor subunit 3; SORD: Sorbitol dehydrogenase; XPO1: Exportin-1; YKT6: Synaptobrevin homolog YKT6; NSF: Vesicle-fusing ATPase; HGS: Hepatocyte growth factor-regulated tyrosine kinase substrate; CHD1L: Chromodomain-helicase-DNA-binding protein 1-like; THOC2: THO complex subunit 2; AGL: Glycogen debranching enzyme; EXOC2: Exocyst complex component 2; KPNB1: Importin subunit beta-1; ROCK2: Rho-associated protein kinase 2; TFRC: Transferrin receptor protein 1; ATP5D: ATP synthase subunit delta; CUL4A: Cullin-4A; ALDH3B1: Aldehyde dehydrogenase family 3 member B1; NUP188: Nucleoporin NUP188; TOP2B: DNA topoisomerase 2-beta; RAB11B: Ras-related protein Rab-11B; PLAA: Phospholipase A-2-activating protein; C8A: Complement component C8 alpha chain; CRNKL1: Crooked neck-like protein 1; GCK: Hexokinase-4; H3: Histone H3; XPO1: Exportin-1; macroH2A1: Core histone macro-H2A.1; NUP107: Nuclear pore complex protein Nup107; TRIP12: E3 ubiquitin-protein ligase TRIP12; NUP214: Nuclear pore complex protein Nup214; RAC1: Ras-related C3 botulinum toxin substrate 1; CSTF3: Cleavage stimulation factor subunit 3; DHX38: Pre-mRNA-splicing factor ATP-dependent RNA helicase PRP16; PWP1: Periodic tryptophan protein 1 homolog; POLR2H: DNA-directed RNA polymerases RPABC3; WDR12: Ribosome biogenesis protein WDR12; UBA5: Ubiquitin-like modifier-activating enzyme 5; PRKAA1: 5′-AMP-activated protein kinase catalytic subunit alpha-1; NSF: Vesicle-fusing ATPase; NUP98: Nuclear pore complex protein Nup98; CTNNBL1: Beta-catenin-like protein 1; GLDC: Glycine dehydrogenase; SUN1: SUN domain-containing protein 1; STAG1: Cohesin subunit SA-1; NOB1: RNA-binding protein NOB1; PIK3R1: Phosphatidylinositol 3-kinase regulatory subunit alpha; SEC24A: Protein transport protein Sec24A; TSFM: Elongation factor Ts; HSPBP1: Hsp70-binding protein 1; CUL1: Cullin-1.

**Table 3:** List of the key immune-related proteins that were specifically identified in the HI-HC group and the LI-LC group, and those shared between both groups. The symbol “−∞” indicates that the protein was detected in the HC group/LC group but not detected in the HI group/LI group. Conversely, “+∞” indicates that the protein was detected in the HI group/LI group but not detected in the HC group/LC group. PPI degree means protein–protein interaction degree.

HI-HC Group (Special Protein)						LI-LC Group (Special Protein)					
Protein ID	Name	PPI Degree	Fold Change	*p*-Value	Regulation	Protein ID	Name	PPI Degree	Fold Change	*p*-Value	Regulation
Endocytosis						Histone					
XP_019954744.1	YKT6	9	+∞	NA	Up	XP_019964890.1	H3	12	+∞	NA	Up
XP_019950084.1	HGS	8	+∞	NA	Up	XP_019941669.1	macroH2A1	11	+∞	NA	Up
XP_019961267.1	TFRC	7	+∞	NA	Up	RNA transport					
XP_019965190.1	RAB11B	6	+∞	NA	Up	XP_019968380.1	NUP107	11	−∞	NA	Down
Complement						XP_019937520.1	NUP214	10	−∞	NA	Down
XP_019955076.1	C8A	6	+∞	NA	Up	XP_019966497.1	NUP98	7	+∞	NA	Up
Other proteins						Other proteins					
XP_019962875.1	ELAVL1	10	+∞	NA	Up	XP_019959917.1	RAC1	10	+∞	NA	Up
XP_019940542.1	ROCK2	7	+∞	NA	Up	XP_019954034.1	POLR2H	9	−2.06	2.10 × 10^−2^	Down
XP_019958157.1	EXOC2	7	+∞	NA	Up	XP_019938010.1	PRKAA1	8	−∞	NA	Down
						XP_019937802.1	PIK3R1	6	+∞	NA	Up
Shared protein											
HI-HC group						LI-LC group					
Protein ID	Name	PPI degree	Fold change	*p*-value	Regulation	Protein ID	Name	PPI degree	Fold change	*p*-value	Regulation
XP_019933706.1	NSF	9	+∞	NA	Up	XP_019933706.1	NSF	8	+∞	NA	Up
XP_019954477.1	CSTF3	10	+∞	NA	Up	XP_019954477.1	CSTF3	9	+∞	NA	Up
XP_019966822.1	XPO1	9	−∞	NA	Down	XP_019966822.1	XPO1	11	+∞	NA	Up

YKT6: Synaptobrevin homolog YKT6; HGS: Hepatocyte growth factor-regulated tyrosine kinase substrate; TFRC: Transferrin receptor protein 1; RAB11B: Ras-related protein Rab-11B; C8A: Complement component C8 alpha chain; ELAVL1: ELAV-like protein 1; ROCK2: Rho-associated protein kinase 2; EXOC2: Exocyst complex component 2.; H3: Histone H3; macroH2A1: Core histone macro-H2A.1; NUP107: Nuclear pore complex protein Nup107; NUP214: Nuclear pore complex protein Nup214; NUP98: Nuclear pore complex protein Nup98; RAC1: Ras-related C3 botulinum toxin substrate 1; POLR2H: DNA-directed RNA polymerases RPABC3; PRKAA1: 5′-AMP-activated protein kinase catalytic subunit alpha-1; PIK3R1: Phosphatidylinositol 3-kinase regulatory subunit alpha; NSF: Vesicle-fusing ATPase; CSTF3: Cleavage stimulation factor subunit 3; XPO1: exportin-1.

## Data Availability

The dataset is available on request from the authors.

## Supplementary Materials

The following supporting information can be downloaded at https://www.mdpi.com/article/10.3390/biology14101417/s1: Figure S1: The molecular weight, peptide counts, peptide length, and peptide coverage distribution of the 3513 proteins; Table S1: A total of 302 DAPs identified in the HI-HC group; Table S2: A total of 317 DAPs identified in the LI-LC group.
