# Supplementary figures and images for: Comparative Proteomics Analysis Reveals Differential Immune Responses of Paralichthys olivaceus to Edwardsiella tarda Infection Under High and Low Temperature
Source: Biology (Basel). 2025 Oct 15;14(10):1417. doi: 10.3390/biology14101417 (PMC12561018; doi:10.3390/biology14101417)

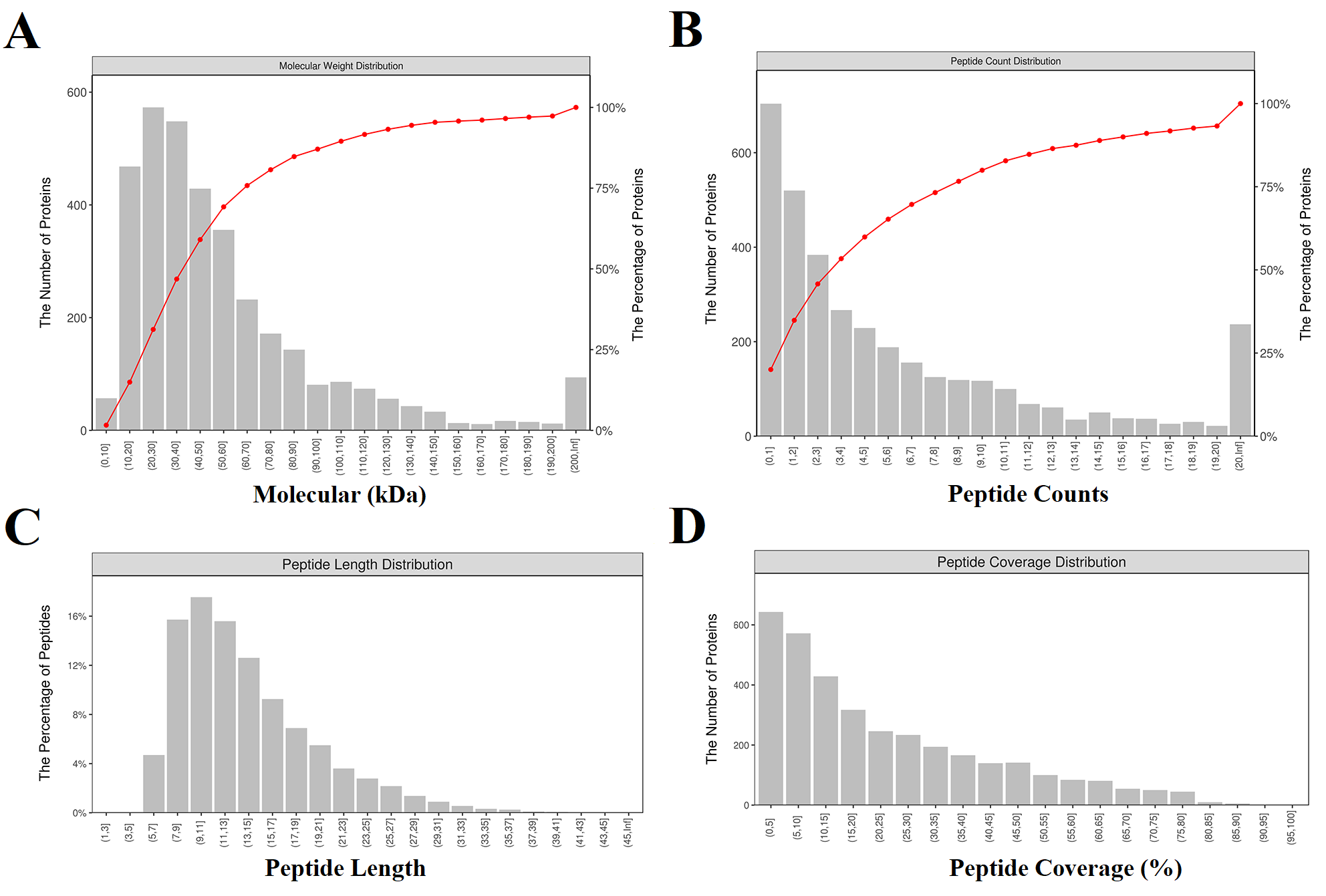

Supplement: Supplementary file 1 [file biology-14-01417-s001.zip › Supplementary File/Figure S1.tif]
